# Comparative Genome-Wide Survey of Single Nucleotide Variation Uncovers the Genetic Diversity and Potential Biomedical Applications among Six *Macaca* Species

**DOI:** 10.3390/ijms19103123

**Published:** 2018-10-11

**Authors:** Jing Li, Zhenxin Fan, Tianlin Sun, Changjun Peng, Bisong Yue, Jing Li

**Affiliations:** 1 Key Laboratory of Bio-Resources and Eco-Environment of Ministry of Education, College of Life Sciences, Sichuan University, Chengdu 610065, Sichuan, China; janelee.nice@gmail.com (J.L.); zxfan@scu.edu.cn (Z.F.); suntianlin23@gmail.com (T.S.); jj-5380682@163.com (C.P.); bsyue@scu.edu.cn (B.Y.); 2 Sichuan Key Laboratory of Conservation Biology on Endangered Wildlife, College of Life Sciences, Sichuan University, Chengdu 610065, Sichuan, China

**Keywords:** SNVs, *Macaca*, macaques, comparative genomics, genetic diversity, biomedical applications

## Abstract

*Macaca* is of great importance in evolutionary and biomedical research. Aiming at elucidating genetic diversity patterns and potential biomedical applications of macaques, we characterized single nucleotide variations (SNVs) of six *Macaca* species based on the reference genome of *Macaca mulatta*. Using eight whole-genome sequences, representing the most comprehensive genomic SNV study in *Macaca* to date, we focused on discovery and comparison of nonsynonymous SNVs (nsSNVs) with bioinformatic tools. We observed that SNV distribution patterns were generally congruent among the eight individuals. Outlier tests of nsSNV distribution patterns detected 319 bins with significantly distinct genetic divergence among macaques, including differences in genes associated with taste transduction, homologous recombination, and fat and protein digestion. Genes with specific nsSNVs in various macaques were differentially enriched for metabolism pathways, such as glycolysis, protein digestion and absorption. On average, 24.95% and 11.67% specific nsSNVs were putatively deleterious according to PolyPhen2 and SIFT4G, respectively, among which the shared deleterious SNVs were located in 564–1981 genes. These genes displayed enrichment signals in the ‘obesity-related traits’ disease category for all surveyed macaques, confirming that they were suitable models for obesity related studies. Additional enriched disease categories were observed in some macaques, exhibiting promising potential for biomedical application. Positively selected genes identified by PAML in most tested *Macaca* species played roles in immune and nervous system, growth and development, and fat metabolism. We propose that metabolism and body size play important roles in the evolutionary adaptation of macaques.

## 1. Introduction

*Macaca* is of great importance in evolutionary and biomedical research, belonging to the Cercopithecidae family, a diverse and widespread primate group that contains 23 extant species [1,2]. These closely related species not only constitute a hotspot in phylogeny research due to their rapid speciation [3], but are also essential nonhuman primate (NHP) models for a wide spectrum of biomedical research [4,5] because of their strong similarities to humans across physiological, developmental, behavioural, immunological, and genetic levels. While rhesus (*M. mulatta*) and cynomolgus (*M. fascicularis*) macaques are the most commonly used NHP models, southern pig-tailed (*M. nemestrina*), Barbary (*M. sylvanus*), Tibetan (*M. thibetana*), Assamese (*M. assamensis*), and Japanese (*M. fuscata*) macaques are emerging models in epidemiology, immunology, neuroscience, pathology, and behaviour science [6,7,8]. Selection of NHP models for biomedical research and evolutionary studies requires clear genetic information about the NHP system [9,10]. While some previous genetic studies have shown that *Macaca* species have considerably diverse genetic backgrounds, displaying enormous heterospecific variation [11,12], and that phenotypic variation exists across geographically distinct individuals [13], genome-wide genetic variations across *Macaca* species have not yet been thoroughly investigated. Additionally, previous biomedical research results and their interpretations can be confounded by indiscriminate use of various macaques, given that genetic data from multiple *Macaca* species could be incorporated into study designs. Therefore, it is necessary to estimate the genome-wide genetic divergence across these macaques.

Single nucleotide variation (SNV) is a primary form of genetic variation in the genome, and is considered to be significantly correlated with various phenotypes including disease susceptibility, illness severity and drug responses [14]. Characterization of SNVs based on whole genome data can provide a comprehensive and thorough dissection of genetic variation. Some genome analysis on *Macaca* species have included the identification of SNVs, but most have focused on evolutionary phylogeny or population genetics [15,16,17,18,19]. To date, only a few studies have analyzed macaque genomic SNVs, but are limited to two flagship *Macaca* species, rhesus and cynomolgus macaques. For example, Malhi et al. [20] identified approximately 23,000 candidate SNVs widely distributed throughout the coding and non-coding regions of *M. mulatta* genome, with large-scale parallel pyrosequencing technology in combination with bioinformatics tools. Also, Ng et al. [21] conducted a comparative study of heterospecific SNVs between human, rhesus and cynomolgus macaque genomes to determine whether macaque alleles are associated with the same phenotypes as their corresponding human alleles. However, a comparable map of genomic SNVs in other macaque species has thus far been lacking.

In this study, we mainly detected, characterized and compared the autosomal heterospecific SNVs of six *Macaca* species, from genome information of eight individuals, with an emphasis on nonsynonymous SNVs (nsSNVs). Employing the rhesus macaque as reference, we describe the genetic diversity patterns and evaluate the genetic differences across *Macaca*. This study aims to not only enrich the genetic data for *Macaca*, but also to facilitate their evolutionary studies as well as inform the selection of optimal NHP models for various research purposes. The identification of functionally significant genetic variations among macaques will open doors for large quantities of downstream studies, which can shed light on gene functions or genetic basis of certain phenotypic traits.

## 2. Results

### 2.1. Genome-Wide Discovery of SNVs

We present the most comprehensive examination of SNVs among macaques to date, involving eight individuals and six species (Table 1) that are of major importance in evolutionary and biomedical research. In total, we identified approximately 10 million final SNVs within each macaque after a series of filtrations described in Fan et al. [22] (detailed results in Table 2). The Chinese rhesus macaque had the smallest number of SNVs, while the southern pig-tailed macaque held the largest number of SNVs, followed by the stumped-tailed macaque (*M. arctoides*) and the Assamese macaque (*M. assamensis*). Counting both homozygous and heterozygous sites, the average SNV frequencies ranged from 3.47/kb (the Chinese rhesus) to 5.15/kb (the southern pig-tailed macaque) for different individuals. 

**Figure 1:**
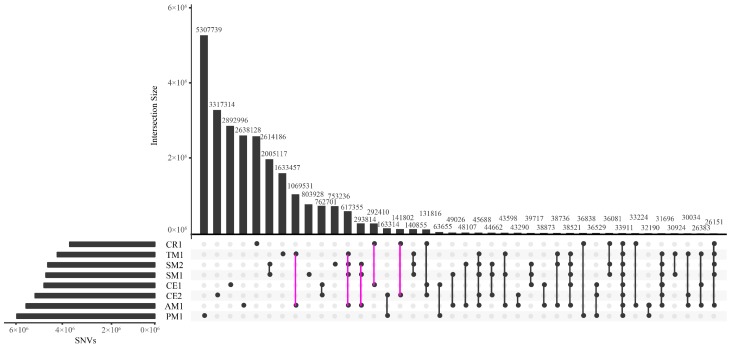
UpSetR plot illustrating the numbers of SNVs shared by different pairs or sets of macaques. Only the first twenty sets are displayed. Intersection Size on the y-axis represents the number of shared SNVs in the pair or set of macaques showed on x-axis.

The numbers of SNVs shared by different macaques or unique to only one macaque are shown in Figure 1, which are generally in accordance with the phylogenetic relationships [26,27]. The Tibetan macaque and Assamese macaque, two closely related species, had more SNVs in common than with other individuals. The second largest SNV set was shared by the Tibetan, Assamese, and stump-tailed macaques, which belong to the same sub-clade of macaques. Finally, belonging to another close species pair, the Chinese rhesus macaque shared more SNVs with the cynomolgus macaques than with others. Approximately 40% SNVs were unique to the southern pig-tailed macaque, which is the most phylogenetically diverged species from rhesus macaque in this survey, representing the largest proportion of species-specific SNVs among our sample set. The Tibetan macaque harboured the least species-specific SNVs with a percentage of 13.68%, mainly due to a less diverse genetic background [22] and high degree of homogeneity, as well as being very closely related to the Assamese macaque.

We analyzed two species that were each represented by two individuals. While the two stump-tailed macaques shared the largest number of SNVs, which indicates very low genetic diversity, the two cynomolgus macaques, originating from Vietnam and Malaysia, had far fewer shared SNVs (Figure 1, Table 2). This lack of common SNVs is even less than those shared between the Tibetan and Assamese macaques, highlighting that the two cynomolgus macaques have strikingly different genetic backgrounds. Therefore, we processed the two cynomolgus macaques separately and surveyed their population-specific SNVs, meanwhile analyzed the two stump-tailed macaques as one sample by only examining the shared SNVs as well as species-specific (not individual-specific) SNVs in subsequent analyses.

The transition/transversion ratios across these macaques fluctuated from 2.17 to 2.25, which is in agreement with the previous studies of human and other primates [28,29], and we observed that C→T and G→A substitutions were most prevalent in the mutation spectrums of *Macaca* species. The heterozygosity level was lowest in the Tibetan macaque (17.16%), followed by two stump-tailed macaques with observed levels equal to 28.76% and 29.32%. These low genetic diversity levels likely resulted from the relatively small effective population sizes of the two species based on the study of Fan et al. [22,25]. In contrast, Chinese rhesus macaque maintained the highest heterozygosity level, followed by Malaysian and Vietnamese cynomolgus macaques, which mirrored the previous finding that these two macaques had high genetic diversity [30]. Heterozygosity estimates revealed varying levels of genetic diversity in our sampled macaques.

### 2.2. Functional Annotation of SNVs

To annotate the putative functional effects of the SNVs detected across the eight macaque genomes, we processed the sites with ANNOVAR [31] (Table 3) and SnpEff [32] (Table S1) based on the rheMac2 reference genome, which produced largely consistent results. Upon mapping our SNVs to the latest rhesus macaque genome assembly Mmul_8 [33] and the human reference genome GRCh37, we also observed concordant ANNOVAR annotations (Table S2). The SNV mapping rates were greater than 99% to Mmul_8 and 77–92% to GRCh37. Though the mapping rate to humans was lower due to larger genetic disparities between human and rhesus macaque, hundreds of additional SNV putative functional variants were obtained. For example, there were 56,515–87,789 exonic SNVs in rheMac2 across these macaques, 56,285–88,136 in Mmul_8, and 59,371–92,402 in human, likely resulting from the more accurate gene models in human genome.

**Figure 2:**
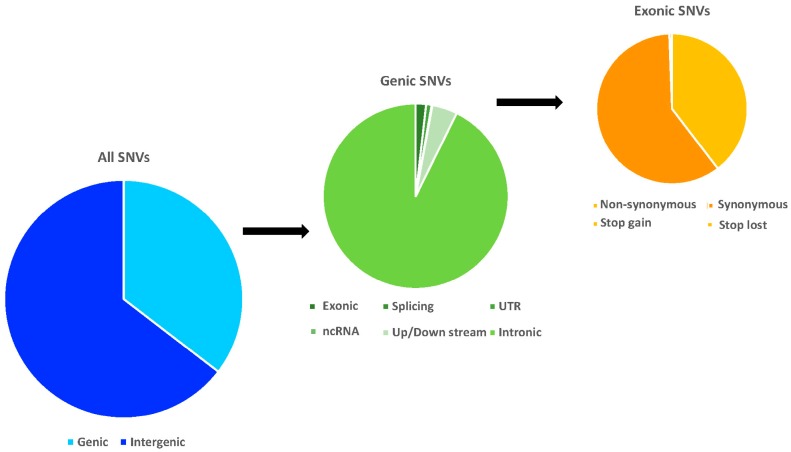
The mean distribution frequencies of the total, genic, and exonic SNVs for all *Macaca* species structurally annotated based on rhesus macaque genome.

Similar to most previous studies [34,35], we consistently observed a higher SNV frequency in intergenic rather than genic regions for all species. As shown in Figure 2, 34.23~36.31% of total SNVs were genic variants, including 0.60~0.69% that were in exons. Among these exonic SNVs, 38.74~40.77% were non-synonymous SNVs (nsSNVs) and less than 1% were stop codon related SNVs (scrSNVs). These nsSNVs and scrSNVs are most likely to be putatively functional variants, which subjected to further analyses later. Finally, the top five mutation biases were shared among all surveyed macaques are as follows: CCG→CCA, AAC→AAT, ACG→ACA, ACA→ACG, and CAC→CAT for codon alterations and A←→T, V←→I, A←→V, P→L, R→H for amino acid changes. An accurate atlas of functional annotations for the SNVs of *Macaca* was drawn based on rhesus macaque and human reference genomes.

### 2.3. Characterization of SNV and nsSNV Distribution Patterns

We investigated the differences in SNV and nsSNV distribution patterns to illustrate their genetic discrepancies among *Macaca* species. Employing non-overlapping 50 kb sliding window scans, we observed that the SNVs were not uniformly distributed on the autosomes. While the distribution patterns were generally congruent among different individuals, a few regions were distinct. These exceptions included thirteen bins with relatively more SNVs across all individuals on chromosomes 3, 4, 5, 9, 10, and 20 (Table S3). It should be noted, these were not problematic regions, as these bins were not located near gaps or incomplete reference genome sequence, nor near regions of high structural variation across individuals found by [36]. Additionally, twelve windows on chromosome 2 exhibited very high SNV densities (9.52–23.74/kb), which displayed only in the Assamese macaque (*M. assamensis*) (Figure S1 and Table S4). The SNV rates in these twelve windows were two to five times higher than the average number of SNVs observed across other windows. Furthermore, seven known genes are located within this SNV-dense region including a highly conserved gene *Robo2* and *Dazl*, *Galnt15*, *Dph3*, *Oxnad1*, *Rftn1*, and *Plcl2*. These genes are involved in immune response, pharmacodynamics, spermatogenesis, O-linked glycosylation, axon guidance and cell migration. While this SNV outlier region could arise due to copy number variations (CNVs) specific to this macaque species, we implemented filtration steps to remove SNVs that overlap with the known duplications in the rhesus macaque [37], rather than that of Assamese macaque using the method described in Fan et al. [22]. Further CNV study on Assamese macaque would be needed to clarify.

**Figure 3:**
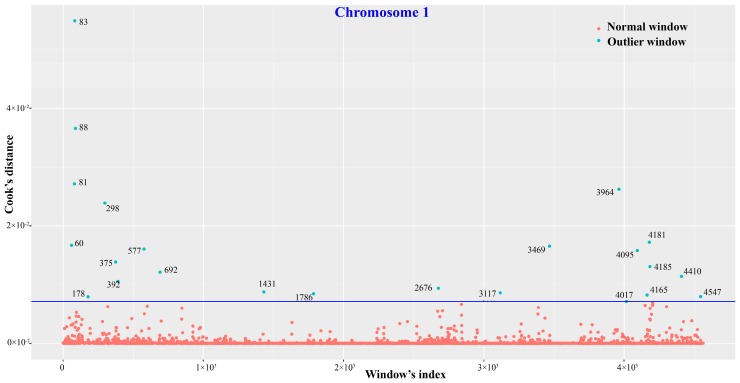
Outlier test of nonsynonymous SNV distribution patterns on chromosome 1 for eight macaque individuals using Cook’s distance test in R. The blue circles represent outlier chromosomal bins that hold significantly more nsSNVs than others. In addition, the numbers next to the blue circles are Cook’s distances of the outlier bins. The dark blue line shows the threshold we used that is 30 times of the mean Cook’s distance.

As for nsSNVs, the overall autosomal distribution patterns were basically congruent across individuals. Outlier tests revealed a total of 319 bins, harbouring 1299 genes with nsSNVs, which displayed remarkably distinct nsSNV distribution patterns across macaques. The outlier test result on chromosome 1 is exhibited in Figure 3, and details of the 319 bins are provided in Table S5.

To ascertain whether genes located in outlier regions of high nsSNV distribution have shared functional roles, we performed enrichment tests for Kyoto Encyclopedia of Genes and Genomes (KEGG) pathways and Gene Ontology (GO) terms for these genes (Table 4). Our results indicate that these genes are linked to metabolism, such as taste transduction (sweet, umami and bitter taste) (mcc04742; *p* = 0.0113), fat digestion and absorption (mcc04975; *p* = 0.0247), pancreatic secretion (mcc04972; *p* = 0.0339), and proteolysis (GO: 0006508; *p* = 0.0200). Genes involved in homologous recombination (HR) (mcc03440; *p* = 0.0163) were also enriched in our outlier regions. Four of the five outlier genes in the HR pathway are associated with double strand break repair (DSBR). Additionally, positive regulation of innate immune response (GO: 0045089; *p* = 0.0448) was one of the enriched GO terms for these outlier genes. The enrichment analysis showed metabolism and immune were two main functional roles for the nsSNVs in outlier windows.

### 2.4. Enrichment Analyses of Specific nsSNVs with Putative Functions

Missense mutations are of special interest since many are believed to have non-marginal functional effects [38]. To further parse the biological meaning of nsSNVs, we conducted enrichment analyses (KEGG pathway, PANTHER, and GO) for specific nsSNVs (results summarized in Table S6). The seven species/populations shared categories associated with metabolism, which emphasizes their metabolic diversity along with the above-mentioned nsSNV distribution patterns. Specifically, genes with specific nsSNVs in the Vietnamese cynomolgus macaque were uniquely depleted for glycolysis (P00024; *p* = 0.0319), the Chinese rhesus macaque for tryptophan metabolism (mcc00380; *p* = 0.0450), the stump-tailed macaque for protein digestion and absorption (mcc04974; *p* = 0.0383) as well as glycosylphosphatidylinositol (GPI)-anchor biosynthesis (mcc00563; *p* = 0.0486), and the Assamese macaque for other glycan degradation (mcc00511; *p* = 0.0439). Additionally, there was an enrichment signal in starch and sucrose metabolism (mcc00500) for both the Vietnamese cynomolgus macaque (*p* = 0.0415) and the southern pig-tailed macaque (*p* = 0.0462). Besides, the homologous recombination pathway (mcc03440; *p* = 0.0233) was also one of the enriched biological pathway terms for the Malaysian cynomolgus macaque, which complemented the above results related to HR and indicated that the Malaysian cynomolgus macaque probably had a distinct homologous recombination function compared with other samples. Also, these macaques shared several significantly enriched biological pathways, including ECM-receptor interaction (mcc04512), hematopoietic cell lineage (mcc04640), ABC transporters (mcc02010) and Fanconi anemia (mcc03460).

Considering that the mammalian target of rapamycin (mTOR) pathway plays a central role in organismal metabolism [39], we investigated the specific nsSNVs within genes belonging to this pathway. There were 10–30 specific nsSNV within 8–22 genes found in these macaques. Tibetan macaque contained a heterozygous SNV in *Map2k2* (ENSMMUT00000027482:exon4:c.G391A:p.V131M) which was quite close to the reported heterozygous mutations causing Cardiofaciocutaneous Syndrome in human, P128Q and G132D [40]. The Tibetan, Assamese, and southern pig-tailed macaques displayed nsSNVs in *Insr* and *Igf1r*, which are key genes in carbohydrates, lipids and protein metabolism as well as growth and insulin-related phenotypes [41,42]. This is consistent with the observations that they are susceptible to diabetes mellitus, and are relatively sturdier than other macaques. 

Significantly enriched GO terms highlighted additional patterns. For example, genes tolerating specific nsSNVs in Malaysian cynomolgus macaque belonged to several immune response related terms (GO:0002218; GO:0002224; GO:0002758; GO:0002764; GO:0002253), which mirrored the findings above based on nsSNV distribution. Also, in the stump-tailed macaque, nsSNVs were enriched in genes linked to the regulation of endocrine process (GO:0044060; *p* = 0.0236). The enrichment survey successfully identified genes with specific nsSNVs in these macaques may indicate different putative functions, and can provide a better understanding of the diversity among *Macaca*.

### 2.5. Putatively Damaging nsSNVs and Their Associated Diseases

Since we were interested in the study of multiple macaque species for potential applications to disease research, we identified the putatively deleterious specific nsSNVs which might be associated with human diseases. The identification was on the basis of the human genome assembly (GRCh37) with PolyPhen2 [43] and SIFT4G [44], since deleterious SNVs in human tend to play vital roles in diseases [45]. There were between 13,175 and 40,283 functional mutations among specific nsSNVs based on prediction of PolyPhen2. On average, 55.90% of these mutations (54.41–57.77%) were assessed to be benign, 16.39% (15.88–16.94%) and 24.95% (22.99–26.82%) were possibly damaging and probably damaging, respectively. Since far fewer deleterious SNVs (9.96–12.86%) were predicted by SIFT4G than PolyPhen2, this indicated the first represented more conservative predictions. The comparison between the two approaches are detailed in Table S7. Enrichment analyses of diseases (KEGG disease, NHGRI GWAS Catalog, and OMIM) were run with KOBAS3.0 [46] for the genes with deleterious specific nsSNVs identified by both programs. Results based on PolyPhen2 prediction are exhibited in Table S8.

The disease phenotypes shared by all macaques here included congenital disorders of development, cardiovascular physiology, skin and soft tissue diseases, obesity-related traits, and immune response, which revealed the major hereditary differences between *Macaca* species and human in the anatomic structure and physiological process. We found that all tested macaques were enriched for nsSNVs in genes linked to type II diabetes mellitus (H00409; OMIM: 125853) except the stump-tailed macaque. In addition, Chinese rhesus macaque is susceptible to type I diabetes (OMIM: 222100; *p* = 0.0085), inferring it may be an ideal model for type I diabetes.

Different macaques showed enrichment signals in many different disease items, reflecting their diversity in disease susceptibility, immune function, and pharmacokinetics. For example, there were “probably damaging” nsSNVs in seven disease-causing genes associated with primary ciliary dyskinesia (PCD) for the stump-tailed macaque, including *Zmynd10*, *Dnah5*, *Dnah11*, *Cenpf*, *Ccdc39*, *Hydin*, and *Rsph1*, a disease which observed to be significantly enriched (H00564; *p* = 0.0042). The genes with “probably damaging” nsSNVs in the Vietnamese cynomolgus macaque were linked to hepatic cysts (HP: 0001407; *p* = 0.0170) and neoplasm of the gastrointestinal tract (HP: 0007378; *p* = 0.0111) according to the g:Profiler enrichment results. There were more drug response related terms that were enriched in the Chinese rhesus macaque gene set than for other macaques, including antineoplastic agent carboplatin (*p* = 0.0008), anti-depressant (*p* = 0.0141) and antipsychotic agents (*p* = 0.0233), amphetamines (0.0481), clozapine (0.0044), methylphenidate (0.0085), statin (*p* = 0.0122), and fenofibrate (*p* = 0.0126).

### 2.6. SNVs Causing Stop Codon Changes

Genes with SNVs causing stop codon changes (scrSNVs) were also investigated by pathway enrichment tests with KOBAS 3.0 [46]. We mainly focused on the disease or medicine related KEGG pathways. A very small proportion of SNVs, ranging from 381 for the Chinese rhesus to 523 for the southern pig-tailed macaque, led to gained or lost termination codons. Due to their induced changes in protein products that could causes serious alterations, we looked closely at these high impact variations. 

The genes with scrSNVs were implicated in the KEGG pathway “drug metabolism cytochrome P450” (mcc00982) in two cynomolgus, stump-tailed and Assamese macaques, including *Fmo2*, *Cyp2d17-like*, *Fmo6p* and *Gsta5* (Figure S2 and Table S9). The scrSNVs were predicted to affect the metabolism of tamoxifen (antitumor hormone drug), cyclophosphamide and ifosfamide, citalopram (anti-depression drug), codeine and morphine (dependence producing drug) for these macaques. Additionally, they were also significantly enriched in the metabolism of cyclophosphamide and ifosfamide (anticarcinogen) for the Malaysian cynomolgus macaque. The four above-mentioned macaques shared two scrSNVs with one in *Fmo2* (ENSMMUT00000027724:exon9:c.C1606T:p.Q536X) and the other in *Cyp2d17-like* (ENSMMUT00000025240:exon1:c.C82T:p.Q28X). FMO2 proteins, which catalyze the oxidation of heteroatom centers in numerous drugs and xenobiotics, of many mammals including rhesus macaque are 536 residues [47]. Here we observed the four macaques produced a 535-amino acid protein due to this nonsense mutation, sharing the same allele found in African Americans and Hispanic populations [48,49], therefore can be good candidate experimental animals for drug studies of these populations. Despite *Cyp2d17-like* is a novel gene, *Cyp2d17*, highly homologous to human *Cyp2d6*, metabolizes human *Cyp2d6* substrates such as bufuralol and dextromethorphan. The previous study showed nonsynonymous variants I297M and N337D in cynomolgus and rhesus *Cyp2d17* significantly altered the catalytic activity of the protein [50]. Therefore, it is possible that the scrSNV in *Cyp2d17-like* can change the drug metabolism of these macaques.

The peroxisome proliferator-activated receptor (PPAR) signalling pathway, which plays a major regulatory role in energy homeostasis and metabolic function [51], emerged in the enriched terms of both the Tibetan and southern pig-tailed macaques (Figure S3). The Tibetan macaque displayed an enrichment signature in PPAR-β/δ that involved three genes with scrSNVs, while the southern pig-tailed macaque showed an enrichment signal in PPAR-γ caused by four genes with scrSNVs. These scrSNVs in the southern pig-tailed macaque might be related to metabolic disease susceptibility according to studies on human and mouse [52,53].

### 2.7. Positive Selection Based on SNVs

Positive selection (also known as Darwinian selection) is an important source of evolutionary innovation and a major force behind the divergence of species [54]. Thus, we surveyed the positively selected genes (PSGs) for *Macaca* species. Of the 11437 single-copy orthologous genes shared between rhesus macaque (*M. mulatta*), the olive baboon (*Papio anubis*), and human (*Homo sapiens*) (see Methods), 12–33 PSGs were identified (FDR, 0.05) for different macaques according to a standard likelihood ratio test (Table 5). The phylogenetic tree of the ten species/populations used as the working topology based on these orthologous sequences is shown in Figure S4. PSGs were generally different for various macaques and the top ranked PSGs were also distinct, confirming that these monkeys diversely adapted to environment under natural selections, even though they were very closely related species.

PSGs for most tested macaques were mainly involved in the nervous system, immunity, growth, development, and fat metabolism. Neuro-related genes were represented in the PSGs of most macaques including the Vietnamese (*Fam53a*) and Malaysian (*Htt*) cynomolgus macaque, the Chinese rhesus macaque (*Myrf*), the Tibetan macaque (*Csrp1*) and the southern pig-tailed macaque (*Htt*, *Ndnf*, and *Tnk2*). PSGs in the Vietnamese cynomolgus macaque (*Fcamr*), the Chinese rhesus macaque (Sharpin), the Assamese macaque (*Myd88*), and particularly the southern pig-tailed macaque (*Cmip*, *Havcr1*, *Prdm1*) also included several innate and adaptive immune related genes. Genes related to growth and development were found to exhibit putatively positive selection patterns in *Macaca* species, including the Vietnam cynomolgus macaque (*Pin1*, *Igfl1*, *Evc2*), the stump-tailed macaque (*Dis3l2*), the Tibetan macaque (*Dis3l2*, Acan), the Assamese macaque (*Kcnk1*, *Ogfr*, *Evc2*) and the southern pig-tailed macaque (*Acan*). In addition, a strong signal of positive selection was found in fat metabolism among all *Macaca* species except the Vietnamese crab-eating macaque and the Chinese rhesus macaque. The four categories of PSGs may reflect some of the main forces driving the species divergence of *Macaca*.

## 3. Discussion

### 3.1. Large Dataset of SNVs for Macaca

We profiled and compared the diversity patterns of genome-wide SNVs for six macaque species by analyzing resequenced, high-coverage genome datasets. Macaques (Cercopithecidae: *Macaca*) are a group of non-human primates of great evolutionary and biomedical importance. Lack of sufficient genomic information has been a significant obstacle for their broader use. Thus, it was necessary to conduct such a systematic variation survey for this group. In comparison to a similar study conducted by Ng et al. [21], our sample set represented more *Macaca* species including the stump-tailed (*M. arctoides*), the Assamese (*M. assamensis*), and the southern pig-tailed (*M. nemestrina*) macaques. These species have received far less survey from a genomic perspective, therefore we included these species in our analyses on macaque SNVs and their putative links to biological functions. Altogether, this study represents the most comprehensive comparative assessment of genomic SNVs for *Macaca* to date.

Our study produced as massive set of genomic differences represented by ten million heterospecific SNVs and had laid the foundation for functional genomic studies in the future with various analyses. This does not just provide a valuable interspecific variation reservoir for *Macaca*, but also facilitates their evolutionary and other studies in the future. The total numbers of SNVs and the SNVs shared by different sets of macaques were generally congruent with their phylogenetic relationships based on previous studies [22,27]. We also provided additional evidence to confirm that Vietnamese and Malaysian crab-eating macaques were quite genetically different, sharing only 43% of their total SNVs, while the two stump-tailed macaques had more than 73% of SNVs in common.

### 3.2. Suggestive Functional Divergence Inferred from SNV Distribution Patterns

SNV distribution patterns among macaques well represent their genetic differentiation. The twelve SNV outlier windows found only in the Assamese macaque (*M. assamensis*) had a unique SNV distribution (Figure S1 and Table S4), and might indicate the unique characteristics of the Assamese macaque in terms of immune response, pharmacodynamics, spermatogenesis, O-linked glycosylation, axon guidance and cell migration. Yet further study is needed to clarify.

Enrichment analysis for the outlier genes based on nsSNV distribution demonstrated that these macaques diverged largely in metabolic pathways. The results in Table 4 suggested that these monkeys may prefer different tastes in food and have discriminating abilities to digest fat and protein, which can give clues to scientific feeding of macaques. The genetic discrepancies in metabolism might also have a connection with the diverse body sizes of these sibling species. For example, the rhesus and cynomolgus macaques are generally slim while the Tibetan, stump-tailed and Assamese macaques look larger and sturdier. This assumption is strengthened by the study of Li et al. [55], which analyzed transcriptomic data and found that the Tibetan macaque had more genes annotated to GO terms related to nutrient reservoir activity than the rhesus macaque, indicating a better ability to store nutrients, contributing to its large body size.

Our enrichment results also indicated that these macaques might have diverging patterns in homologous recombination or specifically DSBR (Table 4). DSBR is essential for maintaining the stability and integrity of genomes and can be used as a potential target of cancer treatment [56,57,58]. If these rhesus monkeys perform DSBR or HR differently, we believe that they tend to react differently to cancer therapy according to the previous studies of DSBR in human and animal models [59,60]. This can provide directions for their potential applications to cancer research.

Finally, the enrichment outputs implied that these macaques differ in innate immune response (Table 4). Given that immune responses of experimental animals heavily affect the results of biomedical experiments, our results confirm that the adverse impacts caused by diverse genetic backgrounds of various macaques should be taken into account in future research designs. On the other hand, their genetic diversity makes it possible to screen optimal models for special research purposes.

### 3.3. Divergent Characteristics Inferred from Enrichment of Putative Functional SNVs

From the perspective of putative functional SNV, this study confirmed that *Macaca* species were generally characterized by a large diversity in metabolism. Genes with specific nsSNVs in different macaques were depleted in multiple metabolic pathways (Table S6), including glycolysis (P00024), protein digestion and absorption (mcc04974), glycan degradation (mcc00511), and starch and sucrose metabolism (mcc00500), which emphasized their metabolic diversity along with the above results of nsSNV distribution pattern analyses. It was quite consistent with the results of SNV distribution patterns (see detailed information about the discrepancies below).

The PPAR pathway was one of the most enriched metabolic pathways for genes with scrSNVs in the Tibetan macaque (PPAR-β/δ) and the southern pig-tailed macaque (PPAR-γ) (Figure S3). In particular, PPAR-γ plays a pivotal role in insulin sensitivity, adipogenesis and placental function [61,62,63]. Mutations in this pathway are responsible for the development of severe insulin resistance, Type-2 diabetes, hypertension, elevated triglycerides and low high-density lipoprotein (HDL) levels, and metabolic syndrome [64]. Combined with the investigation of specific nsSNVs in the mTOR pathway, we infer that the southern pig-tailed macaque is probably susceptible to these metabolism disorders. In contrast, PPAR-β/δ is mainly involved in lipid oxidation and cell proliferation [65,66]. The stop codon mutations and specific nsSNVs in *Insr* and *Igf1r* found in the Tibetan macaque were more likely to contribute to its fat metabolism and comparatively large body size.

Genes with specific nsSNVs in the Malaysian cynomolgus macaque included those associated with activation of innate immune responses and homologous recombination, implying this species probably displays different special innate immune reactions compared to other macaques. These results are also mirrored in our findings based on nsSNV distribution.

Interestingly, one identified gene belonging to the enriched pathway ‘regulation of endocrine process (GO: 0044060)’ for the stump-tailed macaque, *Pomc*, may contribute to the characteristic red face of the stump-tailed macaque, since this gene plays an important role in hair and skin pigmentation based on previous studies [67,68].

### 3.4. Application Potentials in Biomedical Research

Results of this survey suggest that the Chinese rhesus macaque had relatively differential pharmacokinetic characteristics, including responses to antineoplastic agents, anti-depressant and antipsychotic agents, amphetamines and so on. In fact, Irwin et al. reported that the response intensity of α-amphetamine in different NHPs was very different, and it decreased in the order of squirrel monkey > rhesus macaque > pig-tailed macaque > stump-tailed macaque > baboon [69]. This emphasizes that inconsistent results may be generated when using different macaques as experimental animals in drug research. Besides, after examining the scrSNVs in genes belonging to the drug metabolism pathway (mcc00982), we found the assayed cynomolgus, stump-tailed, and Assamese macaques contained a genotype of *Fmo2* also found in African Americans and Hispanic populations [48,49], as well as a nonsense mutation in *Cyp2d17-like*. This indicates that they are very likely to have specific drug-metabolic features as well as potentials to be optimal experimental animals in drug metabolism studies of specific human populations.

All tested macaques can serve as ideal spontaneous NHP models for obesity studies based on our study. For obesity-related traits was one of the enriched disease phenotypes for these macaques (Table S8). This is congruent with the observation that macaques are prone to suffer from obesity [70,71,72], and actually they have been applied to obesity related studies as spontaneous models for a long time [73]. From the aspect of adaptive evolution, we speculated that they were probably more efficient in energy-storing or fat-depositing than humans, in order to survive the harsh environments where availability of food resources always shifts.

Our results also indicated that the Chinese rhesus macaque can be developed as a promising spontaneous model for type I diabetes. Susceptibility to type I diabetes was one of the enriched terms for the Chinese rhesus macaque. Macaques have been wildly used as models for type II diabetes [74,75], which was supported by our convincing results that enrichment signal in type II diabetes appeared for most surveyed macaques. However, the perfect animal model for type I diabetes mellitus has yet to be found [76] and the standardized method of diabetes induction is far from reach [77], which urges the development of additional spontaneous models. Though it still needs more investigation, our survey gives clues to the selection of appropriate macaque models in different types of diabetes studies. The stump-tailed macaque is very likely to be a potentially spontaneous disease model of primary ciliary dyskinesia (PCD) as it displays similar genetic pathogenicity to humans. There was not only a strong enrichment signal identified in PCD, but also probably damaging nsSNVs in the seven disease-causing genes of PCD found in stump-tailed macaque. To date, murine models are most commonly used in PCD studies, and there has been no primate model yet [78]. The stump-tailed macaque may be a strong NHP model to this disease.

Our study also supported there was a genetic basis behind the spontaneous disease susceptibility of crab-eating macaque. This species, along with the rhesus, are the most frequently used NHPs in research, and both are observed to quite often suffer from severely spontaneous diseases in abdominal organs including liver and gastrointestinal tract quite [79]. Our results indicate that Vietnamese cynomolgus macaque had genes with functional SNVs linked to hepatic cysts and neoplasm of the gastrointestinal tract according to the enrichment results, providing a genetic explanation to this disease susceptibility phenomenon.

As in the study of Cornish et al. [80], we also believe that disease enrichments do not guarantee, but reveal a probability that these *Macaca* species harbouring similar loss-of-function variations indicated in human diseases, had a correspondingly higher susceptibility to certain diseases. Our results can lay foundation for developing better NHP models for a wide array of biomedical research.

### 3.5. Positive selections on Macaca Genomes

Previous research found that positive selection shaped the genetic variation of human populations during their earliest settlements in different environments [81,82]. We believe positive selection also heavily impacted the genetic variations of *Macaca* species during their adaptations. Thus, addressing the positive selections across *Macaca* is helpful to understand what drives their genetic differentiation.

Our study found that genes related to nervous system, immune function, growth, development, and fat metabolism display patterns of strong positive selection in most tested macaque species. Our results were in agreement with the previous finding that nervous system [83] and immune response [84,85,86] were two frequent targets for positive selection in primates. Additionally, these different putatively selected genes across these monkeys suggested they probably developed diverse immune mechanisms to adapt to various environments.

Body size is a major discrepancy among our assayed species. The Tibetan macaque is the largest, followed by the stump-tailed and pig-tailed macaques, while the cynomolgus macaque is the smallest. Though the other species are of medium body size, the Assamese macaque is a little more robust than the rhesus macaque. As expected, genes related to growth and development displayed evidence of positive selection, and a strong signal of positive selection was found in fat metabolism genes among all tested *Macaca* species except the Vietnamese eating-crab macaque and the Chinese rhesus macaque, which are relatively leaner species than the others. Along with the aforementioned discussion about metabolic divergences, it implied that body size played an important role in evolutionary adaptation of genus *Macaca*. Also, we agreed that dietary adaptation, including fat metabolism, may have been a major driving force behind species evolution, as was found in previous study [87]. More research needs to conduct to answer questions like when the positive selection occurred and what its functional consequences were.

## 4. Materials and Methods

### 4.1. Genome Data and SNV Calling

Combining public data and in-house resequencing data, we generated a dataset of eight genome sequences of six macaques, including *Macaca mulatta lasiota* (CR1), *M. fascicularis* (CE1, CE2), *M. arctoides* (SM1, SM2), *M. thibetana* (TM1), *M. assamensis* (AM1), and *M. nemestrina* (PM1). Genome data information is listed in Table 1.

Macaque sequence data were mapped to the rhesus reference genome [23] using Bowtie (v2.2.0; [88]) for Hiseq data, using the local alignment algorithm with very sensitive model and proper insert sizes. Default options were used for other parameters. SNVs on autosomes were called individually using Picard (v1.98; http://broadinstitute.github.io/picard/) and GATK (v3.2; [28]). The SOLiD data of CE2 was mapped to rheMac2 with BioScope and called SNVs using the same pipeline. After standard screening procedures, we employed several conservative filtrations to control the data quality with custom Python scripts (scripts are available upon request of the authors). Genome Filters (GF) and Sample Filters (SF) description in [22] were applied to minimize the errors derived from sequencing and alignment, such as errors resulted from triallelic sites, copy number variations, CpG and proximity to indels or other SNVs. We merged the eight SNV files in VCF format with ‘vcf-merge’ [89] to concatenate all SNVs and their genotypes. Sites with missing genotypes in any of the samples were deleted and all callable sites were kept for further analyses.

To identify species- and subspecies-specific SNVs, we screened our results using a custom shell script, using a Guinea baboon (*Papio papio*) as outgroup (GenBank accession: SRX652597 and SRX652598). We mapped the sequences of Guinea baboon to rheMac2 to find SNVs shared by all macaques and baboon, setting mapping threshold as 95%, then excluded them from the species- and subspecies-specific SNVs. For the two individuals of cynomolgus macaques that came from two distinct populations, we identified their population-specific SNVs separately. Upset plot was drawn to show the number of SNVs shared by different pairs or sets of macaques using UpSetR [90].

### 4.2. Functional Annotation of SNVs

Functional annotation was conducted for the final SNV set. To ensure greater accuracy, both ANNOVAR [31] and SnpEff [32] were used with default parameters based on the unique physical positions (bp) of SNVs on chromosomes and the gene annotation from rheMac2. To facilitate further functional analyses, these SNVs were also mapped to the latest assembly of the rhesus genome Mmul_8 and the human reference sequence (GRCh37) using LiftOver (https://genome.ucsc.edu/cgibin/hgLiftOver), and were then structurally characterized with the above two software according to Mmul_8 and GRCh37, respectively. We compared the annotations obtained from the two software on the basis of three different reference genomes. Functional impact of specific nsSNVs based on GRCh37 were predicted with PolyPhen2 [43] and SIFT4G [44] under ‘multiple transcripts’ mode. Classifier model was set as ‘HumDiv’ in PolyPhen2.

### 4.3. Determination of SNV Distribution Patterns and Outlier Detection

We investigated the distribution pattern of SNVs on autosomes by a non-overlapping sliding window approach. The number of SNVs and nsSNVs were counted in each non-overlapping window to draw the distribution patterns. After we tried window sizes of 10 kb, 50 kb, and 100 kb by referring to previous studies [91,92], the size of the window was finally set as 50kb to get a good resolution of the distribution patterns. To figure out whether the SNV-dense bins overlap with regions where the reference genome is questionable or structural variations locate, we examined the bins by compared to ‘bad’ regions found in [36].

Outliers were detected by employing Cook’s distance test in R [93] to identify the windows with significantly different distribution patterns of nsSNVs among the eight macaques, which can infer regions of remarkable genetic differences across the samples/species. A window was identified as an outlier if its Cook’s distance is greater than 30 times of the mean Cook’s distance. The R script was deposited in a GitHub repository (https://github.com/Jing-Li-SCU/macaques_genomic_SNV). Genes with nsSNVs in outlier windows were identified for further analysis.

### 4.4. Functional Enrichment Analyses of SNVs

Enrichment analyses were performed using standalone KOBAS 3.0 [46]. The genes containing nsSNVs in outlier windows, specific nsSNVs, and scrSNVs were respectively subjected to the enrichment tests based on the pathway (KEGG pathway and PANTHER) and GO databases. We set all rhesus genes as the statistical background in the program. Data were statistically analyzed using the hypergeometric test and Benjamini-Hochberg FDR (false discovery rate) correction in KOBAS 3.0. Significant results received *p* values < 0.05. Enriched GO categories were further filtered to Biological Process in GO. Categories with less than three associated genes were discarded.

We also identified enriched disease terms from genes with putatively deleterious nsSNVs predicted by PolyPhen2 and SIFT4G (as described above) using KOBAS 3.0. Terms were considered statistically significant when *p* values < 0.05, too. Furthermore, Human Phenotype Ontology (HPO) enrichment was assessed by g:Profiler [94] for these genes. The number of genes for functional category was set as 5–500, and significance threshold was 0.05 for p value based on the Benjamini-Hochberg FDR. The input genes were compared to a background of all human genes. These analyses allow associating the SNVs of macaques with human diseases and other phenotypic traits, beneficial to understanding the characteristics of macaques and their possibly biomedical applications.

### 4.5. Identification of Positively Selected Genes Based on SNVs

To scan for positively selected genes (PSGs) among the different macaques, we examined 1:1:1 orthologous protein-coding sequences of rhesus macaque (Mmul_8), olive baboon (PapAnu2.0) and human (GRCh37) with OrthoMCL [95]. Large, divergent protein families and proteins with coiled-coil domains were omitted from the analysis. Due to lack of whole genome assemblies, we generated the orthologous coding sequences of other seven macaques by replacing the reference nucleotides with coding SNVs (Mmul_8 version), including *M. mulatta lasiota*, *M. fascicularis* from Vietnam and Malaysia, *M. arctoides*, *M. thibetana*, *M. assamensis*, and *M. nemestrina*. This exploited the fact that gene structures are well-conserved among such closely related species. Then phylogenetic tree, used as the working topology later, of the ten species/populations was reconstructed with RAxML [96] based on these orthologous sequences.

Briefly, CodeML program of PAML (v4.6; [97]) was employed to identify PSGs, with PRANK [98] as the aligner. We tested for selection on each of the eight individual branches within the *Macaca* clade, by comparing branch-site model A (settings: model¼2, NS sites¼2) with null model (settings: fix_omega = 1, omega = 1). Likelihood Ratio Tests (LRTs) were computed and adjusted for multiple testing with a FDR threshold of 0.05. If significant, codon sites with an Empirical Bayes probability 95% and up were considered to be under positive selection. Finally, the function and tissue expressions of these PSGs were manually queried via RhesusBase [99] to dissect the functional differences among these monkeys.

## 5. Conclusions

This study generated a large dataset of heterospecific SNVs for *Macaca*, providing a better understanding of the genetic divergence among macaques. Analyses based on specific nsSNVs demonstrated that these macaques genetically differed in genes belonging to metabolic pathways that include the protein and fat digestion, which might contribute to their variation in body sizes across species.

Analyses of nsSNVs that are putatively deleterious indicated that some macaque species may be valuable for study of numerous diseases. For example, the Chinese rhesus macaque is an ideal candidate for study of type I diabetes, while other macaques may be suitable for type II diabetes research. The Malaysian cynomolgus macaque displayed a distinct pattern in genes associated with homologous recombination and innate immune function, likely indicating its special reaction to cancer therapy and other medical treatments. The stump-tailed macaque may be a putative spontaneous disease model of PCD, while the southern pig-tailed macaque may be vulnerable to metabolic disorders. Different macaques probably have distinct drug-metabolic features and promising application potentials to drug metabolism studies due to tolerating specific nsSNPs and scrSNVs in some drug metabolism key genes. We re-emphasize the importance of genetic variations in model organism’s reactions to biomedical research, and advise that researchers consider the variations when choosing proper macaque models, rather than using different macaques indiscriminately.

Identification of PSGs revealed that genes in pathways associated with immune, neuro system, growth, development, and fat metabolism may have undergone positive selections in *Macaca*. Along with the nsSNV analyses, this result suggests that metabolism and body size played important roles in evolutionary adaptation of this genus.

Although further research is required to thoroughly address some of the issues presented in this study, this survey has not only contributed to the basic understanding of macaque, but has also paved way for their future studies, such as functional genomics studies as well as determination of optimal NHP models in biomedical research.

## Figures and Tables

**Table 1:** Information on genome data.

Scientific Names	Species Symbol	Sample Identifier(s)	GenBank Accession(s)	Sequencing Platform(s)	#Reads	Depth	Total Usable Sites	Sex	Sample Origin(s)	Source(s)
*M. mulatta mulatta*	IR	IR	--	Illumina	20,100,000	5.1X	---	Female	Washington National Primate Research Center	Gibbs et al. 2007 [23]
*M. Mulatta lasiota*	CR	CR1	SRA023856	Illumina	3,299,851,568	45.65X	2,264,143,011	Female	Yunnan, China	Yan et al. 2011 [16]
*M. fascicularis*	CE	CE1	SRA023855	Illumina	3,299,851,568	43.96X	2,245,482,535	Female	Vietnam	Yan et al. 2011 [16]
		CE2	--	SOLiD 3+	3,692,987,634	24.69X	2,261,105,771	Female	Malaysia	Higashino et al. 2012 [24]
*M. arctoides*	SM	SM1	SRX1470574	Illumina	1,001,034,260	34.55X	2,280,352,231	Female	Southwestern China	Fan et al. 2018 [25]
		SM2	SRX1470575	Illumina	471,805,366	20.51X	2,079,812,789	Female	Southwestern China	Fan et al. 2018 [25]
*M. thibetana*	TM	TM1	SRP032525	Illumina	1,275,012,390	36.92X	2,281,638,762	Female	Sichuan, China	Fan et al. 2014 [22]
*M. assamensis*	AM	AM1	SRX1470561	Illumina	1,231,654,664	54.04X	2,011,347,545	Male	Yunnan, China	Fan et al. 2018 [25]
*M. nemestrina*	PM	PM1	SRX1022644	Illumina	770,413,198	25.59X	2,246,079,419	Female	Washington National Primate Research Center	Baylor College of Medicine

-- means there is no GenBank Accession.

**Table 2:** SNV information for each analyzed macaque (see species symbol and sample identifiers in Table 1) including the total number of SNVs, the number of heterozygous (het.) or homozygous (homo.) SNVs, and the number of specific SNVs.

Species Symbol	Sample Identifier(s)	#SNVs	%SNVs	#Homo.	#Het.	%Het.	Ti/Tv	#Specific	%Specific	#Specific Het.
CR	CR1	9,384,359	3.47/kb	3,458,482	5,925,877	63.15	2.23	2,614,186	27.86	2,297,127
CE	CE1	11,751,302	4.35/kb	5,004,945	6,746,357	57.41	2.21	2,892,996	24.62	2,464,551
	CE2	12,000,848	4.44/kb	4,812,493	7,188,355	59.90	2.23	3,317,314	27.64	2,900,657
		5,089,889 ^†^	--	2,712,160	2,377,729	46.71	2.25	762,701	14.98	369,245
SM	SM1	12,712,801	4.69/kb	8,985,648	3,727,153	29.32	2.21	803,928	6.32	740,450
	SM2	11,035,407	4.08/kb	7,861,537	3,173,870	28.76	2.17	753,236	6.83	696,062
		9,353,661 ^†^	--	6,931,659	2,422,002	25.89	2.19	2,005,117	21.43	661,821
TM	TM1	11,937,445	4.42/kb	9,889,106	2,048,339	17.16	2.21	1,633,457	13.68	701,115
AM	AM1	12,249,208	4.52/kb	6,770,425	5,478,783	44.73	2.17	2,638,128	21.54	2,300,208
PM	PM1	13,914,612	5.15/kb	7,613,888	6,300,724	45.28	2.18	5,307,739	38.15	3,860,264

^†^ SNVs shared by two individuals of the same species. -- means not applicable here.

**Table 3:** Functional annotation of (**a**) all processed SNVs and (**b**) exonic SNVs in *Macaca* species based on rheMac2 provided by ANNOVAR.

(**a**)												
	**Total SNVs**	**Intergenic**		**Genic**		**Up/Down Stream**	**Exonic**		**Splicing**	**Intronic**	**UTR**	**ncRNA**
CR1	9,384,359	6,172,058	65.77%	3,212,301	34.23%	144,929	56,515	0.60%	426	2,976,764	28,536	5131
CE1	11,751,302	7,595,956	64.64%	4,155,346	35.36%	181,392	71,807	0.61%	524	3,858,404	37,045	6174
CE2	12,000,848	7,836,824	65.30%	4,164,024	34.70%	189,573	82,821	0.69%	545	3,844,219	39,977	6889
SM	9,353,661	5,956,902	63.69%	3,396,759	36.31%	149,351	63,179	0.68%	411	3,145,443	33,775	4600
TM1	11,937,445	7,642,434	64.02%	4,295,011	35.98%	188,920	77,819	0.65%	519	3,980,620	40,541	6592
AM1	12,249,208	7,880,410	64.33%	4,368,798	35.67%	184,730	73,890	0.60%	505	4,063,127	40,412	6134
PM1	13,914,612	8,944,532	64.28%	4,970,080	35.72%	214,348	87,789	0.63%	612	4,613,059	46,798	7474
(**b**)												
		**Synonymous**				**Nonsynonymous**			**Stop Codon Gain**		**Stop Codon Lost**	
CR1	All	33,295		58.91%		22,839	40.41%		328	0.58%	53	0.09%
	Specific	9576		60.79%		6077	38.58%		92	0.58%	8	0.05%
CE1	All	42,590		59.31%		28,772	40.07%		392	0.55%	53	0.07%
	Specific	10,392		59.50%		6963	39.87%		101	0.58%	10	0.06%
CE2	All	48,535		58.60%		33,769	40.77%		464	0.56%	53	0.06%
	Specific	12,259		59.93%		8091	39.55%		100	0.49%	7	0.03%
SM	All	38,424		60.82%		24,478	38.74%		237	0.38%	40	0.06%
	Specific	7753		61.25%		4839	38.23%		61	0.48%	5	0.04%
TM1	All	45,939		59.03%		31,441	40.40%		375	0.48%	64	0.08%
	Specific	6057		58.76%		4195	40.70%		52	0.50%	4	0.04%
AM1	All	43,945		59.47%		29,497	39.92%		400	0.54%	48	0.06%
	Specific	10,381		60.70%		6599	38.58%		115	0.67%	8	0.05%
PM1	All	51,763		58.96%		35,503	40.44%		466	0.53%	57	0.06%
	Specific	20,034		61.25%		12,507	38.24%		154	0.47%	15	0.05%

**Table 4:** Enrichment outputs of genes with nonsynonymous SNVs in windows with distinct nonsynonymous SNV distribution patterns based on outlier test in R, (**a**) GO term enrichment, (**b**) KEGG pathway enrichment.

(**a**)					
**GO Terms**	**ID**	**Outlier Gene Counts**	**Genome-wide Gene Counts**	***p*** **Value**	**Gene ID**
Proteolysis	GO:0006508	16	151	0.0198	ENSMMUG00000008649, ENSMMUG00000013071, ENSMMUG00000012849, ENSMMUG00000005527, ENSMMUG00000007838, ENSMMUG00000008264, ENSMMUG00000003339, ENSMMUG00000004413, ENSMMUG00000015029, ENSMMUG00000007209, ENSMMUG00000006734, ENSMMUG00000016370, ENSMMUG00000007785, ENSMMUG00000001344, ENSMMUG00000019294, ENSMMUG00000005120
Regulation of proteolysis	GO:0030162	6	40	0.0374	ENSMMUG00000001344, ENSMMUG00000012849, ENSMMUG00000005527, ENSMMUG00000007209, ENSMMUG00000006734, ENSMMUG00000005120
Positive regulation of innate immune response	GO:0045089	3	12	0.0448	ENSMMUG00000019932, ENSMMUG00000003373, ENSMMUG00000008854
Cation transmembrane transporter activity	GO:0008324	12	116	0.0463	ENSMMUG00000031030, ENSMMUG00000015607, ENSMMUG00000006442, ENSMMUG00000018390, ENSMMUG00000013626, ENSMMUG00000030358, ENSMMUG00000007087, ENSMMUG00000032213, ENSMMUG00000004969, ENSMMUG00000007062, ENSMMUG00000007061, ENSMMUG00000010257
(**b**)					
**Pathways**	**ID**	**Outlier Gene Counts**	**Genome-wide Gene Counts**	***p*** **value**	**Gene ID**
Taste transduction	mcc04742	10	70	0.0113	ENSMMUG00000007062, ENSMMUG00000020698, ENSMMUG00000021005, ENSMMUG00000015717, ENSMMUG00000016272, ENSMMUG00000011771, ENSMMUG00000022440, ENSMMUG00000022439, ENSMMUG00000032291, ENSMMUG00000004773
Homologous recombination	mcc03440	5	23	0.0163	ENSMMUG00000007197, ENSMMUG00000003130, ENSMMUG00000022442, ENSMMUG00000014487, ENSMMUG00000019014
Fat digestion and absorption	mcc04975	5	26	0.0247	ENSMMUG00000007692, ENSMMUG00000000825, ENSMMUG00000031036, ENSMMUG00000000148, ENSMMUG00000002724
Pancreatic secretion	mcc04972	9	73	0.0339	ENSMMUG00000020698, ENSMMUG00000031036, ENSMMUG00000018390, ENSMMUG00000015298, ENSMMUG00000010306, ENSMMUG00000000148, ENSMMUG00000021397, ENSMMUG00000032208, ENSMMUG00000002724

**Table 5:** Positively selected genes (PSGs) identified by PAML for different *Macaca* species/population. CE_Viet represents Vietnamese cynomolgus macaque (CE1 in Table 1), and CE_Mal stands for Malaysian cynomolgus macaque (CE2 in Table 1).

Species/Population Symbol	CR	CE_Viet	CE_Mal	SM	TM	AM	PM
Gene Counts	13	14	17	18	12	18	33
Gene Symbol	BRI3 ^†^ KSR1 ZMYND10 PGGT1B SHARPIN EVI2B EP400 RBP3 TBX4 MYRF CHST1 NCKAP5 ^‡^ SEC16B ^‡^	KSR1 ^†^ PIN1 ZNF787DDT IGFL1 FCAMR CCDC33 ^‡^ EVC2 ^‡^ FRMPD2^‡^ SF3B1 FAM53A HAP1 ^‡^ KIAA0825 ZNF474 ^‡^	CMYA5 ^†‡^ BAHD1 EVI2B HTT ^‡^ ZNF646 KIAA1671 ^‡^ KCNMB2 NHLRC1 BAP1 RNF222 SH2D2A DROSHA	ETFB ^†^ ACE SKIV2L BAHD1 EP400 URB2 C9ORF131 PRR14L LMNB1 DIS3L2 KLF13 APOBR ^‡^ ASXL1 ^‡^ DACT2 AIM1 SPAG5 THEM6 RSL24D1	CSRP1 ^†^ VSTM2L LYST ^‡^ DIS3L2 BAHD1 ACAN DDIAS KIAA1549 EXOSC6 THEM6 LYRM5 RSL24D 1	KCNK1 ^†^ AFAP1L1 JSRP1 OGFR INPP5E MYD88 BAHD1 ASPM ^‡^ EVC2 LMNB1 KIF26B^‡^ KIAA1671 ^‡^ GAMT SRRM 2 AQP1 ^‡^ LYST ^‡^ ZNF330 THEM6	HTT ^†‡^, SRRM2 ^‡^ CCDC17 ^‡^, WHAMM BDP1 ^‡^, ALPK3 ^‡^ NDNF, ACAN ^‡^ TRIM28, LYST ^‡^ LRRC10B, RARRES1 ^‡^ CMIP, RCSD1 ERCC6 ^‡^, TFG GAMT, KIF26B ^‡^ SLC9C2, NAT6 DHRS9, EXO1 ^‡^ HAVCR1, TNK2 ^‡^ DDX31, XIRP2 ^‡^ WDR73, HVCN1 ^‡^ TMEM126B ^‡^, APOBR, NDUFV3 PRDM1, YWHAE

^†^ Gene symbol in bold represents the top PSG for each species/population. ^‡^ PSGs with probably damaging nsSNVs.

## Supplementary Materials

Supplementary materials can be found at http://www.mdpi.com/1422-0067/19/10/3123/s1.

## Abbreviations

SNV	Single nucleotide variation
NHP	Nonhuman primate
CNV	Copy number variation
A	Adenine
C	Cytosine
G	Guanine
T	Thymine
A	Alanine
T	Threonine
V	Valine
I	Isoleucine
P	Proline
L	Leucine
R	Arginine
H	Histidine
KEGG	Kyoto encyclopedia of genes and genomes
GO	Gene ontology
DSBR	Double strand break repair
HR	homologous recombination
PCD	Primary ciliary dyskinesia
ECM	Extracellular matrix
mTOR	Mammalian target of rapamycin
PPAR	Peroxisome proliferator-activated receptor
HDL	High-density lipoprotein
PSG	Positively selected gene
PAML	Phylogenetic analysis by maximum likelihood
