# Supplementary material for: Comparative Genome-Wide Survey of Single Nucleotide Variation Uncovers the Genetic Diversity and Potential Biomedical Applications among Six Macaca Species
Source: Int J Mol Sci. 2018 Oct 11;19(10):3123. doi: 10.3390/ijms19103123 (PMC6212917; doi:10.3390/ijms19103123)
Supplement: Supplementary file 1 [file ijms-19-03123-s001.zip › supplementary/Fig S1-4.pdf]

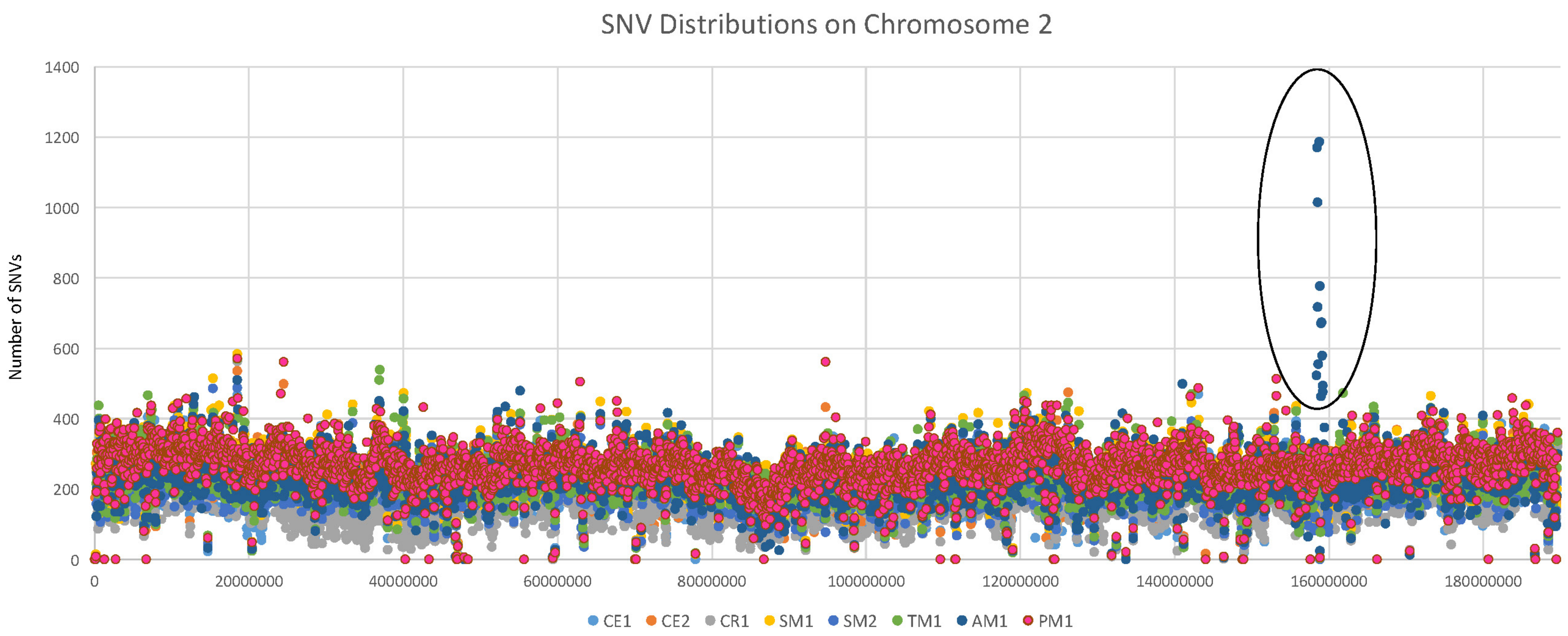

Figure S1. The twelve windows on chromosome 2, labelled in the circle, of Assamese macaque (M. assamensis) exhibiting very high SNV frequency.

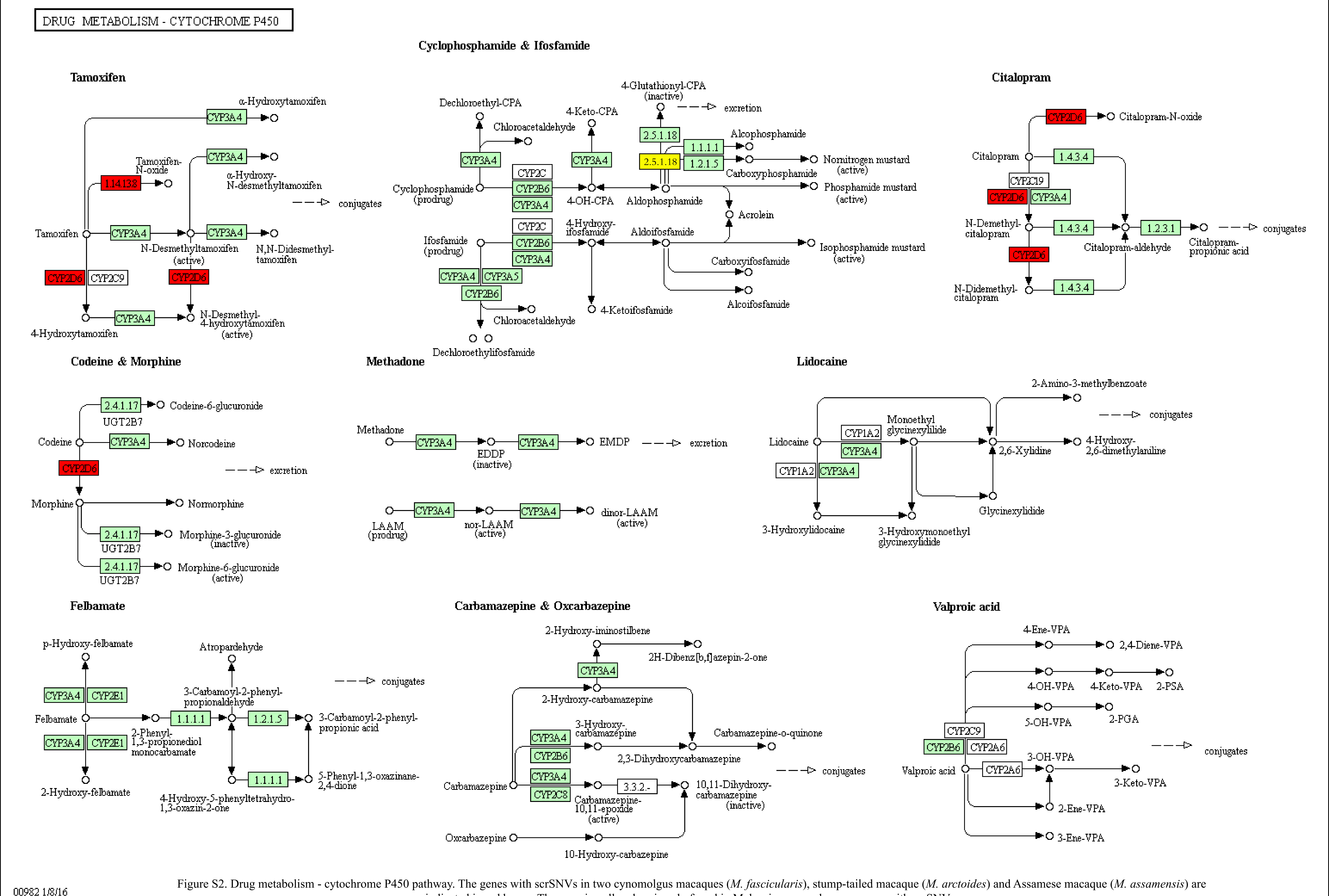

Figure S2. Drug metabolism - cytochrome P450 pathway. The genes with scrSNVs in two cynomolgus macaques (*M. fascicularis*), stump-tailed macaque (*M. arctoides*) and Assamese macaque (*M. assamensis*) are indicated in red boxes. The gene in yellow box is only found in Malaysia cynomolgus macaque with scrSNVs.

(c) Kanehisa Laboratories

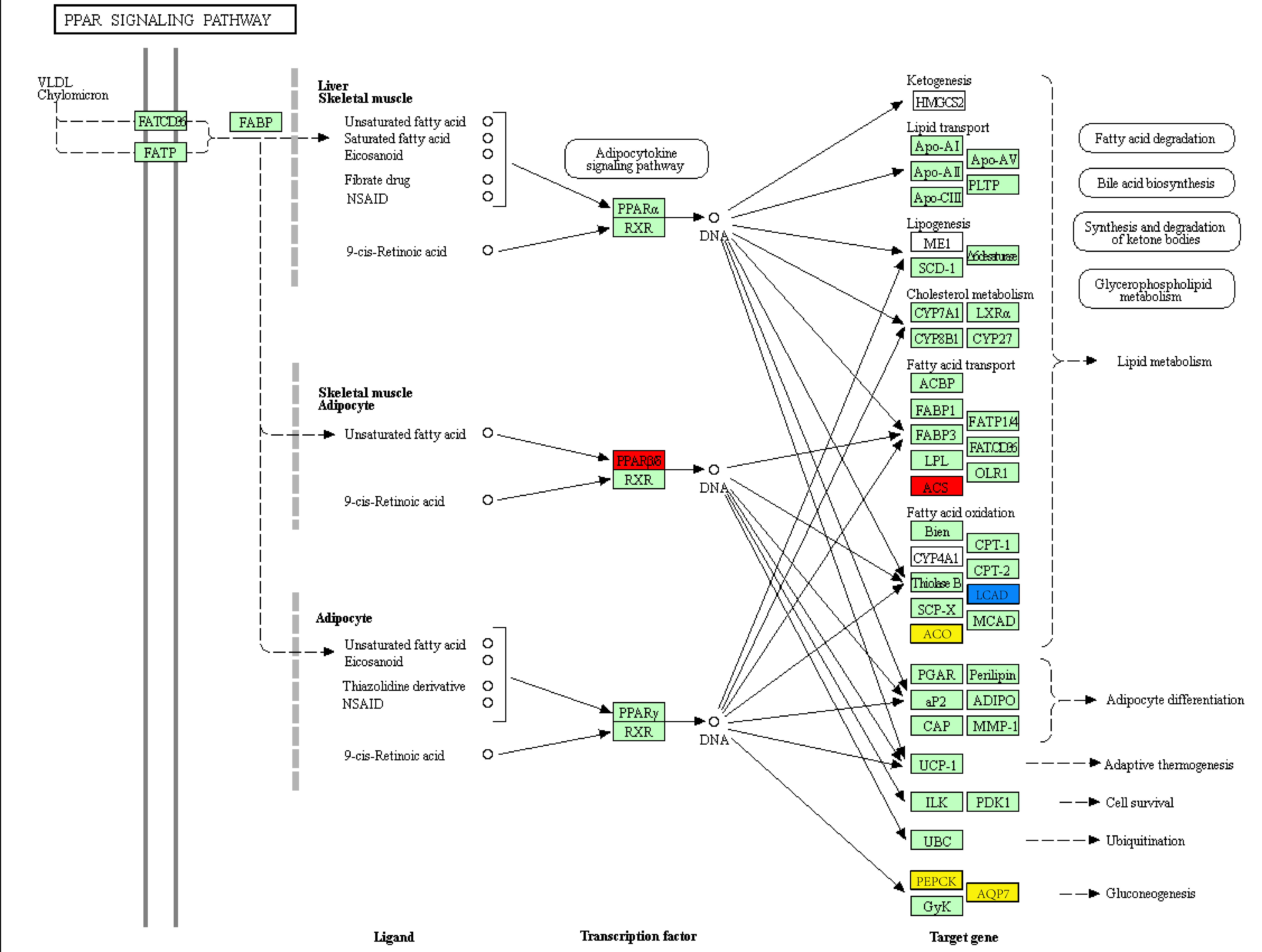

03320 6/3/16
(c) Kanehisa Laboratories

Figure S3. PPAR signaling pathway. The genes with scrSNVs for Tibetan macaque (*M. thibetana*) are indicated in red boxes. And these in southern pig-tailed macaque (*M. nemestrina*) are labelled in yellow. The gene with scrSNVs shared by two species is labelled in blue box.

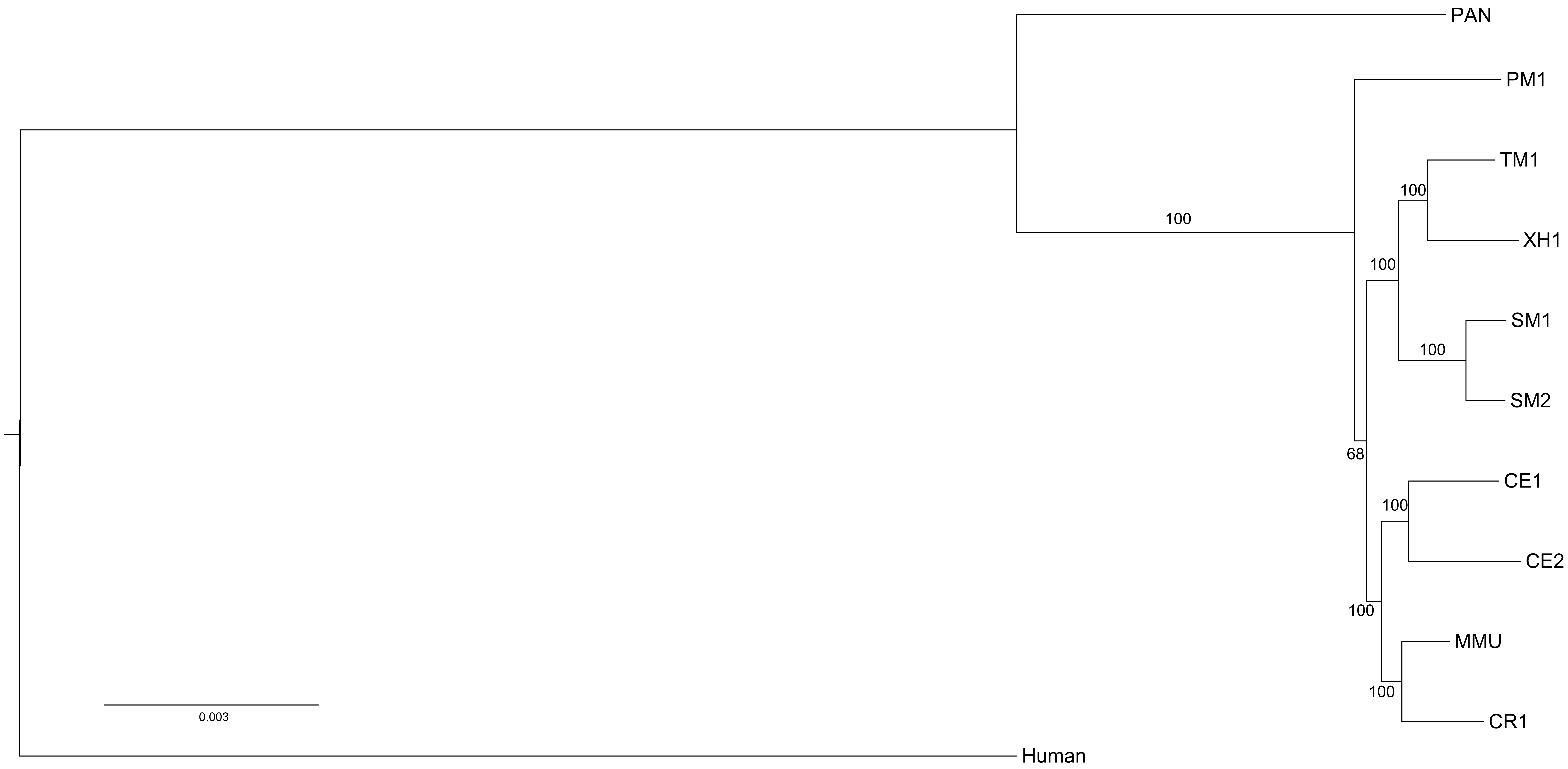

Figure S4. Phylogenetic tree of the Macaca species/populations, the olive baboon and human was reconstructed with RAxML based on the orthologous sequences, used as the working topology for positive selection analysis.
