# Supplementary material for: Comparative Genome-Wide Survey of Single Nucleotide Variation Uncovers the Genetic Diversity and Potential Biomedical Applications among Six Macaca Species
Source: Int J Mol Sci. 2018 Oct 11;19(10):3123. doi: 10.3390/ijms19103123 (PMC6212917; doi:10.3390/ijms19103123)
Supplement: Supplementary file 1 [file ijms-19-03123-s001.zip › supplementary/Table S1-2 S6-9-revision.docx]

**Table S1** Outputs of SnpEff for eight individuals in *Macaca*. (a) Annotations for all SNVs, (b) annotation of SNVs with special interests.

(a)

| Type | CR1 | CE1 | CE2 | SM | TM1 | AM1 | PM1 |
| --- | --- | --- | --- | --- | --- | --- | --- |
| 3_prime_UTR_variant | 23425 | 30691 | 32424 | 26690 | 32750 | 32171 | 38315 |
| 5_prime_UTR_premature  _start_codon_gain_variant | 1173 | 1550 | 1757 | 1606 | 1756 | 1898 | 2137 |
| 5_prime_UTR_variant | 7519 | 9450 | 10936 | 10067 | 11190 | 11678 | 12450 |
| downstream_gene_variant | 698368 | 885226 | 894181 | 713448 | 915928 | 890586 | 1045062 |
| initiator_codon_variant | 8 | 9 | 7 | 6 | 8 | 8 | 8 |
| intergenic_region | 6316750 | 7777092 | 8025895 | 6106084 | 7831051 | 8064907 | 9158573 |
| intragenic_variant | 3063546 | 3969475 | 3969234 | 3244117 | 4101284 | 4179698 | 4750289 |
| intron_variant | 2991467 | 3877476 | 3865358 | 3162340 | 4001306 | 4082478 | 4636435 |
| missense_variant | **22813** | **28751** | **33757** | **24467** | **31412** | **29483** | **35485** |
| non_canonical_start_codon | 0 | 1 | 0 | 1 | 1 | 1 | 0 |
| non_coding_exon_variant | 5115 | 6162 | 6888 | 4594 | 6576 | 6131 | 7452 |
| non_coding_transcript_variant | 3067372 | 3973954 | 3974451 | 3247408 | 4106091 | 4184068 | 4755732 |
| splice_acceptor_variant | 240 | 293 | 345 | 246 | 319 | 295 | 348 |
| splice_donor_variant | 285 | 337 | 343 | 253 | 339 | 315 | 411 |
| splice_region_variant | 7279 | 9446 | 10416 | 8252 | 9985 | 9525 | 11438 |
| start_lost | 42 | 40 | 44 | 26 | 43 | 35 | 48 |
| stop_gained | 328 | 392 | 464 | 237 | 375 | 400 | 466 |
| stop_lost | 53 | 53 | 53 | 40 | 64 | 48 | 57 |
| stop_retained_variant | 28 | 35 | 37 | 45 | 47 | 48 | 59 |
| synonymous_variant | 33611 | 42963 | 49011 | 38758 | 46340 | 44320 | 52202 |
| upstream_gene_variant | 713626 | 903247 | 913105 | 728844 | 936605 | 910499 | 1065758 |

(b)

| Samples | Missense variants | Stop gain variants | Stop lost variants | Specific missense variants | Specific stop gain variants | Specific stop lost variants |
| --- | --- | --- | --- | --- | --- | --- |
| **CR1** | 22813 | 328 | 53 | 6071 | 92 | 8 |
| **CE1** | 28751 | 392 | 53 | 6933 | 100 | 10 |
| **CE2** | 33757 | 464 | 53 | 8083 | 100 | 7 |
| **SM** | 24467 | 237 | 40 | 4835 | 61 | 5 |
| **TM1** | 31412 | 375 | 64 | 4185 | 52 | 4 |
| **AM1** | 29483 | 400 | 48 | 6593 | 115 | 8 |
| **PM1** | 35485 | 466 | 57 | 12492 | 154 | 15 |

**Table S2** Comparison of SNV annotations derived from ANNOVAR and SnpEff based on RheMac2, MMUL8 and GRCh37.

|  | Total SNV | | | Exonic | | | Nonsynonymous | | | Specific Nonsynonymous | | |
| --- | --- | --- | --- | --- | --- | --- | --- | --- | --- | --- | --- | --- |
|  | **rheMac2** | **MMUL8** | **GRCh37** | **rheMac2** | **MMUL8** | **GRCh37** | **rheMac2** | **MMUL8** | **GRCh37** | **rheMac2** | **MMUL8** | **GRCh37** |
| CR1 | 9,384,359 | 9,306,988 | 8,356,460 | 56,515 | 56,285 | 59,371 | 22,839 | 21,797 | 19,270 | 6,077 | 6,309 | 7,190 |
| CE1 | 11,751,302 | 11,659,949 | 9,304,595 | 71,807 | 71,622 | 62,837 | 28,772 | 28,311 | 21,401 | 6,963 | 7,258 | 7,008 |
| CE2 | 12,000,848 | 11,891,243 | 10,780,168 | 82,821 | 83,438 | 87,484 | 33,769 | 33,829 | 29,409 | 8,091 | 7,725 | 9,403 |
| SM | 9,353,661 | 9,292,144 | 8,613,287 | 63,179 | 63,506 | 67,579 | 24,478 | 24,519 | 20,172 | 4,839 | 5,244 | 5,798 |
| TM1 | 11,937,445 | 11,844,704 | 10,668,704 | 77,819 | 77,948 | 82,040 | 31,441 | 31,326 | 25,674 | 4,195 | 4,469 | 4,852 |
| AM1 | 12,249,208 | 12,162,587 | 11,162,246 | 73,890 | 73,784 | 78,180 | 29,497 | 29,368 | 25,364 | 6,599 | 6,952 | 7,653 |
| PM1 | 13,914,612 | 13,811,481 | 12,500,137 | 87,789 | 88,136 | 92,402 | 35,503 | 35,695 | 30,718 | 12,507 | 13,419 | 14,855 |

[**Table S3**](file:///F:\lab_meeting_20180518-Jing\Table%20S3.xlsx) SNV frequencies in 13 highly diverse regions for all tested macaques.

[**Table S4**](file:///F:\lab_meeting_20180518-Jing\Table%20S4.xlsx) SNV frequencies in 12 highly diverse regions only found in Assamese macaque (*M. assamensis*).

[**Table S5**](file:///F:\lab_meeting_20180518-Jing\Table%20S5.xlsx) Details of windows with distinct nsSNV distribution patterns according to the outlier test with R.

**Table S6** Significantly enriched terms of (a) Gene Ontology (GO) and (b) pathways for genes with specific nonsynonymous SNVs in Macaca species.

(a)

| CR1 | cyclase activity  phosphorus-oxygen lyase activity  cyclic nucleotide biosynthetic process  cyclic purine nucleotide metabolic process  cAMP biosynthetic process | GO:0009975  GO:0016849  GO:0009190  GO:0052652  GO:0006171 | p = 0.0216  p = 0.0309  p = 0.0423  p = 0.0423  p = 0.0425 |
| --- | --- | --- | --- |
| CE1 | **serine-type endopeptidase inhibitor activity**  **regulation of axon guidance**  **regulation of axon extension involved in axon guidance**  **neuron projection extension involved in neuron projection guidance**  **axon extension involved in axon guidance**  **regulation of axonogenesis**  **endopeptidase inhibitor activity**  **cofactor metabolic process**  **axon extension**  **neuron projection extension**  **regulation of axon extension**  **semaphorin-plexin signaling pathway**  **regulation of extent of cell growth**  **tRNA processing**  **endopeptidase regulator activity**  **peptidase inhibitor activity**  **regulation of developmental growth** | **GO:0004867**  **GO:1902667**  **GO:0048841**  **GO:1902284**  **GO:0048846**  **GO:0050770**  **GO:0004866**  **GO:0051186**  **GO:0048675**  **GO:1990138**  **GO:0030516**  **GO:0071526**  **GO:0061387**  **GO:0008033**  **GO:0061135**  **GO:0030414**  **GO:0048638** | **p = 0.0125**  **p = 0.0246**  **p = 0.0246**  **p = 0.0246**  **p = 0.0246**  **p = 0.0319**  **p = 0.0365**  **p = 0.0365**  **p = 0.0383**  **p = 0.0383**  **p = 0.0383**  **p = 0.0383**  **p = 0.0383**  **p = 0.0398**  **p = 0.0433**  **p = 0.0433**  **p = 0.0450** |
| **CE2** | activation of innate immune response  ncRNA metabolic process  pattern recognition receptor signaling pathway  toll-like receptor signaling pathway  innate immune response-activating signal transduction  immune response-regulating signaling pathway  preribosome  activation of immune response | GO:0002218  GO:0034660  GO:0002221  GO:0002224  GO:0002758  GO:0002764  GO:0030684  GO:0002253 | p = 0.0226  p = 0.0371  p = 0.0373  p = 0.0373  p = 0.0373  p = 0.0435  p = 0.0460  p = 0.0474 |
| **SM** | endocrine process  3'-5' DNA helicase activity  regulation of endocrine process | GO:0050886  GO:0043138  GO:0044060 | p = 0.0236  p = 0.0340  p = 0.0340 |
| **TM1** | None |  |  |
| **AM1** | hydrolase activity, hydrolyzing O-glycosyl compounds  homophilic cell adhesion via plasma membrane adhesion molecules  regulation of cell growth  cell adhesion molecule binding | GO:0004553  GO:0007156  GO:0001558  GO:0050839 | p = 0.0339  p = 0.0347  p = 0.0430  p = 0.0439 |
| **PM1** | carboxypeptidase activity  microtubule cytoskeleton  exopeptidase activity | GO:0004180  GO:0015630  GO:0008238 | p = 0.0279  p = 0.0299  p = 0.0376 |

(b)

| CR1 | Fanconi anemia pathway  ECM-receptor interaction  ABC transporters  One carbon pool by folate  Tryptophan metabolism | mcc03460  mcc04512  mcc02010  mcc00670  mcc00380 | KEGG pathway  KEGG pathway  KEGG pathway  KEGG pathway  KEGG pathway | p = 7.426e-05  p = 0.0013  p = 0.0070  p = 0.0185  p = 0.0450 |
| --- | --- | --- | --- | --- |
| **CE1** | ECM-receptor interaction  Hematopoietic cell lineage  Glycolysis  Starch and sucrose metabolism  Non-homologous end-joining  ABC transporters | mcc04512  mcc04640  P00024  mcc00500  mcc03450  mcc02010 | KEGG pathway  KEGG pathway  PANTHER  KEGG pathway  KEGG pathway  KEGG pathway | p = 0.0004  p = 0.0073  p = 0.0319  p = 0.0415  p = 0.0450  p = 0.0486 |
| **CE2** | Fanconi anemia pathway  ECM-receptor interaction  Homologous recombination | mcc03460  mcc04512  mcc03440 | KEGG pathway  KEGG pathway  KEGG pathway | p = 0.0081  p = 0.0106  p = 0.0233 |
| **SM** | ECM-receptor interaction  Fanconi anemia pathway  ABC transporters  Protein digestion and absorption  Glycosylphosphatidylinositol(GPI)-anchor biosynthesis | mcc04512  mcc03460  mcc02010  mcc04974  mcc00563 | KEGG pathway  KEGG pathway  KEGG pathway  KEGG pathway  KEGG pathway | p = 0.0028  p = 0.0037  p = 0.0300  p = 0.0383  p = 0.0486 |
| **TM1** | ECM-receptor interaction  ABC transporters  Fanconi anemia pathway  One carbon pool by folate  Butanoate metabolism  Hematopoietic cell lineage | mcc04512  mcc02010  mcc03460  mcc00670  mcc00650  mcc04640 | KEGG pathway  KEGG pathway  KEGG pathway  KEGG pathway  KEGG pathway  KEGG pathway | p = 0.0003  p = 0.0064  p = 0.0136  p = 0.0280  p = 0.0281  p = 0.0298 |
| **AM1** | ECM-receptor interaction  Fanconi anemia pathway  Hematopoietic cell lineage  ABC transporters  Blood coagulation  Other glycan degradation | mcc04512  mcc03460  mcc04640  mcc02010  P00011  mcc00511 | KEGG pathway  KEGG pathway  KEGG pathway  KEGG pathway  PANTHER  KEGG pathway | p = 0.0005  p = 0.0009  p = 0.0030  p = 0.0127  p = 0.0344  p = 0.0439 |
| **PM1** | Complement and coagulation cascades  Fanconi anemia pathway  ABC transporters  ECM-receptor interaction  Hematopoietic cell lineage  Aminoacyl-tRNA biosynthesis  Starch and sucrose metabolism  Butanoate metabolism | mcc04610  mcc03460  mcc02010  mcc04512  mcc04640  mcc00970  mcc00500  mcc00650 | KEGG pathway  KEGG pathway  KEGG pathway  KEGG pathway  KEGG pathway  KEGG pathway  KEGG pathway  KEGG pathway | p = 0.0080  p = 0.0088  p = 0.0144  p = 0.0228  p = 0.0360  p = 0.0385  p = 0.0462  p = 0.0466 |

**Table S7** Comparison of putatively deleterious specific nsSNVs identified by PolyPhen2 and SIFT4G.

| **Samples** | **#Annotations** | | **#Deleterious SNVs**^†^ | | **%Deleterious SNVs** | | **#Shared deleterious SNVs** |
| --- | --- | --- | --- | --- | --- | --- | --- |
|  | PolyPhen2 | SIFT4G | PolyPhen2 | SIFT4G | PolyPhen2 | SIFT4G |  |
| **CR1** | 19235 | 15032 | 4798 | 1753 (176) ^‡^ | 24.94 | 11.66 | 731 |
| **CE1** | 19195 | 17370 | 5067 | 2234 (240) ^‡^ | 26.40 | 12.86 | 801 |
| **CE2** | 25645 | 20645 | 6464 | 2532 (183) ^‡^ | 25.21 | 12.26 | 945 |
| **SM** | 15776 | 11991 | 3627 | 1194 (106) ^‡^ | 22.99 | 9.96 | 433 |
| **TM1** | 13175 | 10551 | 3314 | 1278 (118) ^‡^ | 25.15 | 12.11 | 575 |
| **AM1** | 20670 | 16007 | 5544 | 1974 (177) ^‡^ | 26.82 | 12.33 | 807 |
| **PM1** | 40283 | 30953 | 9338 | 3254 (244) ^‡^ | 23.18 | 10.51 | 1077 |

^†^ mean ‘probably damaging SNVs’ for PolyPhen2

^‡^numbers of SNVs which can be found in dbSNP

**Table S8** Outputs of disease enrichment with KOBAS 3.0 for genes harboring probably damaging specific nsSNVs identified by PolyPhen2.

1. **The enriched phenotypes shared by all macaques:**

height and body mass index

congenital disorders of development

cardiovascular physiology

skin and soft tissue

obesity-related traits

immune response to smallpox

1. **Enriched terms related to metabolism diseases**

Type 2 diabetes: CR1, CE1, CE2, TM1, AM1, PM1

Diabetes, type 1, susceptibility to I (OMIM: 222100)：CR1

Congenital disorders of amino acid metabolism: CE1, CE2, TM1

Uric acid levels: CR1, SM

Liver enzyme levels (alkaline phosphatase): SM, AM1

Vitamin D levels: CE1, PM1

Urate levels in obese individuals: CE1, CE2, CR1, SM

Coenzyme Q10 deficiency (KEGG DISEASE: H00999): CE2

Circulating myeloperoxidase levels (serum): CE2, PM1

Primary hyperammonemic disorders (KEGG DISEASE: H01398): CE2

Homocysteine levels: CR1, SM

Folate pathway vitamin levels: SM, AM1

Cholesterol (total, HDL, LDL): CR1, SM, TM1, AM1, PM1

Congenital disorders of ion transport and metabolism: SM

Metabolite levels (5-HIAA/ MHPG Ratio): TM1

Plasma omega-6 polyunsaturated fatty acid levels (arachidonic acid): CE2, CR1, TM1, AM1, PM1

Glycerophospholipid levels: TM1, PM1

Phospholipid levels: TM1, AM1, PM1

Fatty acid levels: AM1, PM1

Very long-chain saturated fatty acid levels (fatty acid 22:0): AM1

Aluminium levels: SM, TM1, AM1,

Manganese levels: CE1, CE2, AM1

Magnesium levels: CE1, CR1

Endocrine and metabolic diseases: CE1, CR1, AM1, PM1

1. **Enriched terms related to nervous and sensory system diseases**

Nervous and sensory system diseases: CE1, CE2, SM, TM1, AM1, PM1

Neurodegenerative diseases: CE1, SM, TM1, AM1, PM1

Alzheimer's disease: CE1, CE2, TM1, AM1, PM1

Major depressive disorder: CE1, CE2, SM, TM1, AM1, PM1

Parkinson's disease (KEGG DISEASE: H00057): SM, AM1, CE1, TM1, PM1

Bipolar disorder: CE1, CE2, CR1, SM, TM1, AM1, PM1

Distal hereditary motor neuropathies (dHMN) (KEGG DISEASE: H00856): CE1, SM

Attention deficit hyperactivity disorder: CE1, CR1, PM1

Spinocerebellar ataxia (SCA) (KEGG DISEASE: H00063): CE1, CE2, SM, TM1, AM1

Hippocampal atrophy: CE2, SM

Hippocampal sclerosis: CR1

Post-traumatic stress disorder (asjusted for relatedness): CR1

Amyloid A serum levels: SM

Deafness, autosomal recessive (autosomal recessive) (KEGG DISEASE: H00605): CE2, CR1, SM, AM1, TM1, PM1

Macular degeneration (KEGG DISEASE: H00821): CE2, CR1, TM1, AM1, PM1

Retinitis pigmentosa (RP) (KEGG DISEASE: H00527): CE2

Myopia (pathological): SM, TM1, AM1

Corneal structure: CE1, CE2, TM1

Refractive astigmatism: CE1, SM

Glaucoma (primary open-angle): CE1, CE2, CR1, SM

Age-related nuclear cataracts: CE1, SM, PM1

Glaucoma (primary open-angle): CE1, CE2, CR1, SM

Congenital stationary night blindness (CSNB) (KEGG DISEASE: H00787): SM

1. Enriched terms related to skeletal and muscular system

Musculoskeletal diseases: CE1, CE2, CR1, TM1, AM1, PM1

Congenital muscular dystrophies (CMD/MDC) (KEGG DISEASE: H00590): CR1, TM1, AM1

Bone mineral density: CE1, CR1, SM, AM1, PM1

Amyotrophic lateral sclerosis (sporadic): CE1, CE2, CR1, SM, AM1, PM1

Myasthenia gravis: CE2

Metaphyseal dysplasias (KEGG DISEASE: H00479): CR1

Ankylosing spondylitis: TM1

Adolescent idiopathic scoliosis: CR1,

1. Enriched terms related to circulatory system

Cardiovascular diseases: CE1, CE2, SM, TM1, AM1, PM1

Coronary artery calcification: CE1, SM

Fanconi anemia (KEGG DISEASE: H00238): CE2, CR1, AM1

Hematologic diseases: CE1, CE2, SM, TM1, AM1, PM1

Leukemia, acute myeloid (OMIM: 601626)/Acute lymphoblastic leukemia: CE1, TM1, AM1, PM1

Cardiac diseases: CE1, CE2, SM, AM1

Stroke: CE1, TM1

Hypertension, essential (OMIM: 145500): CE1, CR1, PM1

PR interval: CR1, SM

Atrial fibrillation (KEGG DISEASE:H00731): CE2, SM

Cardiac hypertrophy: TM1, AM1

Blood trace element (Cu levels): TM1, AM1

Dilated cardiomyopathy (DCM) (KEGG DISEASE: H00294): CE2, AM1

Hereditary spherocytosis (KEGG DISEASE: H00230): PM1

1. Enriched terms related to digestive system

Inflammatory bowel disease (IBD) (KEGG DISEASE: H01227): CE1, CR1, SM, AM1, PM1

Ulcerative colitis: CE1, TM1

Helicobacter pylori serologic status: CE2

Colorectal cancer (diet interaction): CE2

1. Enriched terms related to immune system

Immune system diseases: CE1, CE2, CR1, TM1, PM1

Allergies and autoimmune diseases: CE2, CR1, TM1, PM1

Immune response to measles-mumps-rubella vaccine: CE2

HIV-1 control: TM1

Complement regulatory protein defects (KEGG DISEASE: H00106): TM1

Primary immunodeficiency: TM1

Interferon alpha levels in systemic lupus erythematosus: PM1

HIV-1 susceptibility: CE1

Lupus nephritis in systemic lupus erythematosus: SM, TM1, AM1

Allergic dermatitis: CE1, CE2, CR1, SM, PM1

1. Enriched terms related to drug responds

Adverse response to chemotherapy (neutropenia/leucopenia) (carboplatin): CR1, SM, TM1, AM1, PM1

Response to taxane treatment (placlitaxel ): CR1, SM

Clozapine-induced cytotoxicity: CR1, CE1, CE2, AM1

Response to antineoplastic agents: SM, AM1, CE2, PM1

Response to methylphenidate treatment in attention-deficit/hyperactivity disorder: CR1, PM1

Response to statin therapy(LDL cholesterol change): CE1, CR1, AM1, PM1

Diisocyanate-induced asthma: CE1, CE2, SM, TM1, AM1, PM1

Response to fenofibrate (adiponectin levels): CR1, TM1

Response to anti-retroviral therapy (ddI/d4T) in HIV-1 infection HIV: CE1, CE2, TM1

Response to anti-depressant treatment in major depressive disorder: CE1, CR1,

Response to antipsychotic /antidepressants treatment: CR1, SM, AM1

Response to amphetamines: CR1

Response to serotonin reuptake inhibitors in major depressive disorder: SM

Response to simvastatin treatment (PCSK9 protein level change):  SM

Triptolide cytotoxicity: TM1

Epilepsy and lamotrigine-induced maculopapular eruptions: TM1

Response to zileuton treatment in asthma (FEV1 change interaction): AM1

Adverse response to chemotherapy in breast cancer (alopecia) (cyclophosphamide+doxorubicin+/-5FU): CR1, PM1

1. Enriched terms related to other diesease

Primary microcephaly (KEGG DISEASE:H00269): CE1, CE2, SM, TM1, PM1

Nephronophthisis-medullary cystic kidney disease (KEGG DISEASE: H00537): CR1, TM1, PM1

Idiopathic membranous nephropathy: CE1

Seckel syndrome (KEGG DISEASE: H00992): CE1, CE2, SM, TM1, AM1, PM1

Hair morphology: CE2, CR1, AM1, PM1

Multiple sclerosis: CE1, AM1, PM1

Psoriasis: CE2

Melanoma: CE2, AM1, PM1

Vitiligo: CR1

Endometriosis: CE1

Menarche (age at onset): CE1, CE2, CR1, SM, TM1, AM1

Congenital disorders of DNA repair systems: CE1, CE2

Airway responsiveness in chronic obstructive/ pulmonary disease: CE2, SM, TM1

Airflow obstruction: SM, TM1

Emphysema imaging phenotypes: CR1, TM1, AM1

Pulmonary function:  TM1, AM1

Chronic bronchitis and chronic obstructive pulmonary disease: TM1

Alcohol dependence (OMIM:103780): CE2

Cancers of the urinary system and male genital organs: CE2

Primary ciliary dyskinesia (KEGG DISEASE: H00564): CE2, SM, TM1

D-dimer levels: CR1, AM1, PM1

Thyroid dyshormonogenesis (KEGG DISEASE: H00251): SM

Cannabis use (initiation): TM1

Malaria: TM1

Rheumatoid arthritis: TM1

Hepatitis C induced liver fibrosis: TM1

Thyroid hormone levels: TM1

Epidermolysis bullosa (OMIM 226650; KEGG DISEASE: H00586): TM1

Mitochondrial DNA depletion syndrome (MDS) (KEGG DISEASE: H00469): PM1

Disorders of nucleotide excision repair (KEGG DISEASE: H00403): CE1, CE2, PM1

**Table S9 Information on the scrSNVs in enriched genes belonging to KEGG pathway “drug metabolism - cytochrome P450” (mcc00982).**

| **Samples** | **Input number** | **Background number** | **P values** | **Enriched genes** | **Gene symbols** | **scrSNVs** |
| --- | --- | --- | --- | --- | --- | --- |
| **CE1** | 3 | 48 | 0.0236 | ENSMMUG00000019727,  ENSMMUG00000017962, ENSMMUG00000007956 | FMO2  CYP2D17-like  FMO6P | ENSMMUT00000027724:exon9:c.C1606T:p.Q536X,  ENSMMUT00000025240:exon1:c.C82T:p.Q28X  ENSMMUT00000011129:exon8:c.C1435T:p.R479X |
| **CE2** | 3 | 48 | 0.0318 | ENSMMUG00000019727, ENSMMUG00000017962,  ENSMMUG00000017137 | FMO2  CYP2D17-like  GSTA5 | ENSMMUT00000027724:exon9:c.C1606T:p.Q536X  ENSMMUT00000025240:exon1:c.C82T:p.Q28X  ENSMMUT00000024724:exon2:c.G115T:p.E39X |
| **SM** | 3 | 48 | 0.0080 | ENSMMUG00000019727, ENSMMUG00000017962, ENSMMUG00000007956 | FMO2  CYP2D17-like  FMO6P | ENSMMUT00000027724:exon9:c.C1606T:p.Q536X  ENSMMUT00000025240:exon1:c.C82T:p.Q28X  ENSMMUT00000011129:exon5:c.G761A:p.W254X |
| **AM1** | 3 | 48 | 0.0297 | ENSMMUG00000019727, ENSMMUG00000017962, ENSMMUG00000007956 | FMO2  CYP2D17-like  FMO6P | ENSMMUT00000027724:exon9:c.C1606T:p.Q536X  ENSMMUT00000025240:exon1:c.C82T:p.Q28X  ENSMMUT00000011129:exon8:c.T1618C:p.X540Q |
